# Facing Complexity: Outcomes of Surgical Bailouts from Complications of Transcatheter Aortic Valve Replacement in a Single High-Volume Center

**DOI:** 10.3390/jcm14093051

**Published:** 2025-04-28

**Authors:** Andrea Daprati, Andrea Garatti, Marco Guerrini, Antonio Sisinni, Luca Arzuffi, Federico Soma, Carlo de Vincentiis, Lorenzo Menicanti

**Affiliations:** 1 Cardiac Surgery Unit, IRCCS Policlinico S. Donato, 20097 Milan, Italy; andrea.garatti@grupposandonato.it (A.G.); federico.soma@grupposandonato.it (F.S.); carlo.devincentiis@grupposandonato.it (C.d.V.); lorenzo.menicanti@grupposandonato.it (L.M.); 2 Clinical and Interventional Cardiology Department, IRCCS Policlinico S. Donato, 20097 Milan, Italy; marco.guerrini@grupposandonato.it (M.G.); antonio.sisinni@grupposandonato.it (A.S.); luca.arzuffi@grupposandonato.it (L.A.)

**Keywords:** transcatheter aortic valve replacement, aortic valve replacement, cardiac surgery

## Abstract

**Background:** Transcatheter aortic valve replacement (TAVR) is at the forefront of structural heart programs all over the world. With a growing number of TAVR procedures in lower-risk and younger patients, acute and chronic complications require decisive treatment. The aim of the present study is to retrospectively analyze the efficacy of surgical bailout strategies in case of complications from TAVR that had been performed in the highest-volume center in Italy over the past ten years. **Methods:** Acute complications were defined as events occurring in the first 24 h after surgery, while chronic complications were defined as events occurring within the first year post-implant. We retrospectively analyzed the 2731 patients who had undergone TAVR at our institution from January 2015 to August 2024. **Results:** A total of 21 patients were included, with a median age of 78 years (IQR 11y). The majority of patients underwent TAVR with a self-expanding prosthesis (76%). A total of 11 patients (52%) presented acute complications, of which the most common were aortic dissection (*n* = 4 [19%]) followed by left ventricular perforation (*n* = 3 [14%]). The most common chronic complication was early endocarditis (*n* = 5 [24%]). The most common bailout strategy was aortic valve replacement (AVR), which was sufficient in 10 patients (48%), followed by complete root replacement (*n* = 4 [19%]). In-hospital mortality was higher in acute compared with chronic complications albeit not statistically significant (*n* = 4 [36%] vs. *n* = 2 [20%], *p* = 0.64), highlighting the very high risk of all these surgeries. **Conclusions:** Bailout and post-TAVR surgery are critical issues, with overall acceptable yet significant mortality considering the very high risk of these procedures. In our experience, half of the overall complications cannot be resolved with a simple explant and subsequent valve replacement, thereby underlining the importance of skilled cardiothoracic surgery teams on site to address complex issues such as ventricular perforation and emergency aortic/root replacement.

## 1. Introduction

In the last decade, therapeutic options for the treatment of aortic valve disease, in particular of degenerative aortic stenosis, have evolved exponentially since the widespread introduction of transcatheter devices. With the increasing number of endovascular heart interventions, transcatheter aortic valve replacement (TAVR) is at the forefront of structural heart programs all over the world [1].

Alongside the technological advancements in this field comes a degree of technical expertise from interventional cardiologists and cardiac surgeons alike to provide a minimally invasive transcatheter solution for aortic valve disease, even in patients with more comorbidities and more complex peripheral vessel anatomy. Since their approval for use in lower-risk and younger patients, the incidence of these procedures has surpassed traditional surgery in terms of raw numbers [2]. The most recent European guidelines recommend TAVR instead of surgery for high-risk and suitable moderate-risk patients as well as in patients aged over 75 years [3]. Surgical aortic valve replacement (SAVR) still has higher recommendation for younger and low-risk patients mainly because the durability of transcatheter heart valves (THV) is still the subject of several ongoing studies [4].

Over the past years, we have observed a significant learning curve with TAVR, with undoubtable improvements over time in the rates of procedural success and reduction in overall complications, as well as the need for surgical bailout.

Complication rates of TAVR mostly involve the access site [5], which, in the vast majority of cases, is the femoral axis; however, the most feared adverse events during transcatheter procedures are the ones requiring a surgical bailout strategy, among which there are aortic annular rupture, iatrogenic aortic dissection, and the perforation of one of the cardiac chambers [6]. The management of complications in these patients is challenging both in terms of acuity of presentation and potential rapid deterioration following the adverse event and in terms of postoperative course and management in a population of elderly individuals who often present a higher frailty index and comorbidities that precluded them from open surgery in the first place [7].

All these life-threatening complications require immediate bailout in the majority of cases, thereby needing an expert cardiac surgery team available for immediate response to provide even the most complex and timely solutions for potentially devastating events with rates of in-hospital mortality as high as 50% [8].

In regard to non-emergent SAVR after TAVR, mortality increases by 15% compared with primary SAVR. This increase is often attributed to the need for more extensive and challenging surgeries of major complexity, including aortic root replacement (which is required in nearly one-third of patients), and to patient frailty. With increased invasiveness and complexity, these surgeries are often characterized by longer and more challenging management in their postoperative course.

Moreover, the rate of explanted TAVR devices has increased remarkably over the past few years, highlighting the importance of accurate patient selection in order to achieve outcomes comparable with those of traditional surgery [9]. In addition to this, it is evident that in a large percentage of cases, a simple aortic valve replacement is not enough, thereby requiring more extensive surgery to address the aortic root and the ascending aorta [10].

Throughout the literature, most of the studies assessing the complications of TAVR or evaluating explants after the procedure are based on data from clinical trials or large registries [11]. Because the outcomes from these papers include either intraprocedural data or retrospective incidences of device explants, it is rare that all types of events requiring surgical bailout both in the acute and in the chronic setting are included in the same population. Moreover, because of the scarcity of complete reports of these complications and the scattered nature of the types of bailout interventions, there is no consensus on the behavior that should be followed by the cardiothoracic (CT) surgeons involved.

The aim of the present study is to retrospectively analyze the efficacy and the short-term outcomes of surgical bailout strategies in case of complications arising in patients undergoing TAVR that had been performed in the highest-volume center in Italy over the past ten years.

## 2. Materials and Methods

At our institution, transcatheter aortic valve replacement has been widely adopted as a treatment option for patients with aortic valve disease since the C.E. approval of the earliest devices. The overall number of procedures is on the rise, with up to 400 TAVR devices implanted yearly at our center.

We retrospectively analyzed all the patients who had required surgical bailout or reintervention due to complications arising within one year of undergoing transcatheter aortic valve replacement at our institution in the last ten years: from January 2015 to August 2024. Preoperative, intraprocedural, and postoperative variables were obtained from in-hospital charts and operative notes. A total of 2731 patients had undergone TAVR at our institution during the study period, of which 316 (11.6%) received a balloon-expandable prosthesis and 2415 (88.4%) received a self-expanding prosthesis. A total of 21 patients (0.77%) were included in the retrospective analysis, as they required surgical bailout from major complications during the procedure or within a year after TAVR. Preoperative risk assessment was performed via the STS score, and all patient cases were discussed in a Heart Team setting prior to the decision of undergoing TAVR.

Complications following TAVR were defined as intraprocedural or post-procedural events requiring the prompt intervention of the CT surgery department. Of these, acute complications were defined as events occurring within the first 24 h after surgery, while chronic complications were defined as events occurring in the first year post-implant, which were of three types: infective endocarditis (defined according to the modified Duke criteria), valve dysfunction (defined according to the VARC-3 criteria), and evidence of either subclinical thrombosis of the device or significant perivalvular leak (PVL). Patients who required cardiac surgery bailout in the acute setting were retrieved from OR records, while those who underwent surgery within the first year post-implant were retrieved from hospital admission or discharge notes. There are no missing data and no biases of selection, as all the patients and hospital notes were screened for the inclusion criteria of surgical bailout following TAVR. The normality distribution of the continuous variables was tested and validated with the Shapiro–Wilk test. Results were presented as mean values ± standard deviation or median and interquartile range. Parametric (one-way analysis of variance) and non-parametric (U Mann–Whitney) tests were used to compare continuous variables. The χ2 test or the Fisher’s exact test was used to compare categorical variables. A *p*-value <0.05 was considered statistically significant. Potential predictors of 30-day mortality and major morbidity rates were tested with stepwise multivariable logistic regression analysis. Only factors with a *p*-value <0.05 were considered significant, and the corresponding odds ratio with the 95% confidence interval (CI) was reported. Calibration of the model was assessed with the Hosmer–Lemeshow goodness-of-fit test, and the area under the curve was calculated with the receiver operating curve analysis. All statistical analyses were performed with the help of GraphPad Prism (GraphPad Software, Prism 9.5.1, 225 Franklin Street. Fl. 26, Boston, MA 02110, USA).

## 3. Results

The 21 patients that were analyzed had a median age of 78 years (IQR 11y), and of these, 9 (43%) were male. Degenerative calcific aortic stenosis and bioprosthetic valve degeneration were the two most common indications for the primary TAVR procedure (14 [67%] and 4 [19%], respectively). For patients aged under 75 years (6 [29%]), the main driver for the choice of transcatheter aortic valve replacement was significant frailty or reduced life expectancy. Two patients were over 70 years old and presented with bioprosthetic valve degeneration alongside patent bypass grafts and were therefore deemed at a very high risk for repeat surgery. For two patients, after the Heart Team discussion, TAVR was chosen over SAVR because of reduced life expectancy with a concomitant diagnosis of malignant tumor. In one case, TAVR was chosen because of severe comorbidities such as end-stage renal disease and severe COPD on home oxygen therapy. In another case, TAVR was chosen over the surgical option in a young patient with extreme obesity (BMI = 44 kg/m^2^), prior bioprosthetic aortic valve replacement, and who was denied bariatric surgery because of the severity of the aortic stenosis due to the degeneration of the tissue valve.

**Figure 1:**
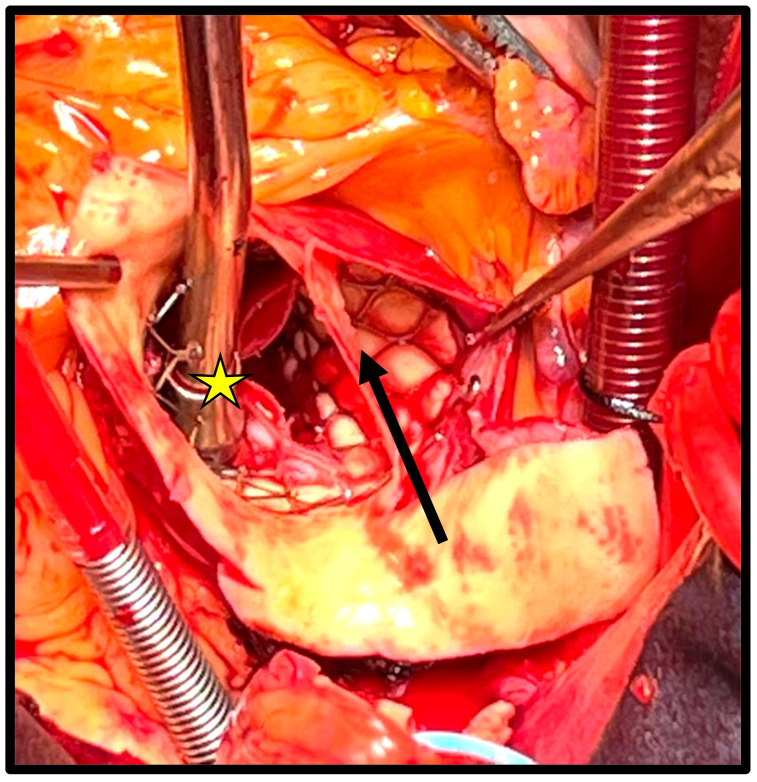
Acute aortic dissection of the aortic root and ascending aorta following implant of a self-expanding TAVR and originating from the outflow stent. The black arrow shows the dissection flap. The yellow star above the suction device shows the correct position of the TAVR.

The majority of patients underwent TAVR with a self-expanding prosthesis (16 [76%]), and the rate of complications when comparing the two types of devices was not statistically significant (*p* = 0.09). A total of 11 patients (52%) presented acute complications, of which the most common were aortic dissection (*n* = 4 [19%], Figure 1) and left ventricular perforation (*n* = 3 [14%]). The most common chronic complication was early endocarditis (*n* = 5 [24%]), followed by early prosthetic valve dysfunction (*n* = 3 [14%]); both complications were assessed according to the VARC-3 criteria.

The most common bailout strategy was aortic valve replacement (AVR), which was sufficient in 10 patients (48%), and, in particular, it was sufficient for all those patients with recent implant of the bioprosthesis and issues related to valve function only, followed by complete aortic root replacement (i.e., Bentall procedure) in 4 (19%) patients, with overall comparable mortality regardless of the surgical procedure performed. In-hospital mortality was higher in acute compared with chronic complications, albeit not statistically significant (*n* = 4 [36%] vs. *n* = 2 [20%], *p* = 0.64). Mortality in the balloon-expandable group was 60% (*n* = 3) and 19% (*n* = 3) in the self-expanding group. The comparison of mortality for the two types of TAVRs was not statistically significant (*p* = 0.11), despite the phenomenon of annular rupture in balloon-expandable devices, which carries a 0% survival rate in our population. Logistic regression analysis identified preoperative age (OR, 1.16; 95% CI, 1.01–1.30; *p* = 0.014), acute complications (OR, 2.35; 95% CI, 1.91–4.56; *p* = 0.002), and aortic annulus rupture (OR, 6.58; 95% CI, 2.56–8.67; *p* = 0.001) as independent risk factors for 30-day mortality (Hosmer–Lemeshow: χ^2^ = 9.27, *p* = 0.45; area under the curve, 0.78; 95% CI, 0.62–0.86). A summary of all the patients’ characteristics and results can be found in Table 1 and Table 2.

## 4. Discussion

Bailout and post-TAVR surgery have become critical issues, especially since the approval of transcatheter devices in younger and lower-risk individuals. It is well documented that procedures requiring the explant of TAVR devices are on the rise [12] and often with much more complex surgeries than simple aortic valve replacement. Given that a vast number of patients undergoing transcatheter aortic valve replacement are considered at a higher risk or with higher frailty, every complication requiring the intervention of the CT team should be thoroughly evaluated. Fortunately, the rate of complications requiring intervention of the CT surgery team is extremely low, even in experienced centers. This likely reflects a “safe” learning curve, in which the expertise of the center increases, and it allows more and more complex procedures.

From our series, and throughout Europe, the majority of patients who undergo bailout surgery for complications arising from TAVR are either in their seventies or eighties or present significant comorbidities or technical risks that would have represented significant deterrents from traditional surgery. In our population, the majority of patients were female, and for whom the underlying disease and reason for undergoing TAVR was degenerative calcific aortic stenosis.

In our experience, and in accordance with data from the literature [12], half of the overall complications (both acute and chronic) cannot be resolved with a simple explant and subsequent valve replacement, highlighting the variety of clinical scenarios that can play out from complications of transcatheter aortic valve replacement. The interaction of these devices with the aortic root makes the process of explant a potentially life-threatening challenge, even in the most expert hands. This further supports the recommendation that transcatheter aortic valve replacement should only be done in settings where immediate support by an expert CT surgery team is readily available. We have observed in our population that there is no difference in terms of mortality for the various types of surgical procedures, highlighting that in a center where complex aortic root surgery and reoperations are performed routinely, even challenging bailouts can be accomplished with acceptable survival. With decreasing numbers of surgical aortic valve replacements and increasing TAVR devices, the expertise of surgeons at managing the complications arising from transcatheter procedures can only be acquired with large surgical volumes. Therefore, patients should be referred to specialized tertiary facilities with the expertise required for potential complications.

The paucity of events requiring surgical bailout can be attributed to catastrophic events, regardless of the preoperative assessment. Moreover, based on our experience as well as on published reports, some considerations can be added. First, given that transfemoral TAVRs are nowadays nearly always performed under local anesthesia, we recommend that in cases with anticipated anatomically complex issues (peripheral vessels with severe tortuosity, peripheral access with severe calcifications, extreme horizontal ascending aorta, hostile root with small sinuses and/or low coronary ostia), a Heart Team discussion suggesting general anesthesia should be performed. In case of the need for a bailout TAVR procedure (shockwave of femoral access, severe aortic or arch tortuosity requiring the “buddy wire” technique with a super stiff parallel wire, coronary artery protection, or occurrence of post-TAVR deployment sinus sequestration) if hemodynamic instability should occur, this preparation can avoid time-consuming induction of a general anesthesia that could lead to progressive development of cardiogenic shock and potentially even cardiac arrest. Furthermore, from the anatomical viewpoint, a preoperative CT scan workup of the aortic root allows selection of the proper TAVR device according to its exclusion criteria. In case of severe calcific bulging of the aortic annulus, asymmetrical calcifications of the mitro-aortic continuity, or LVOT, balloon-expandable TAVR should be excluded, given the higher risk of annulus rupture in this setting. On the other hand, a very horizontal ascending aorta or a significant discrepancy between the larger annulus and the small aortic root or sino-tubular junction should be considered more complex anatomical features for a self-expanding TAVR, as any misalignment of the prosthesis could increase the risk of non-coronary sinus tearing and development of aortic rupture or aortic dissection.

Regarding surgical timing, it is evident that acute catastrophic complications require emergent surgical correction. In our study, surgical timing together with hemodynamic instability were the two major determinants of extremely higher mortality in this group of patients. In the cases of pericardial effusion and/or heart structure perforation due to the guidewire, pericardial drainage and careful clinical monitoring were enough to stabilize the patient and to waive or delay surgical correction in a more stable condition. Conversely, aortic rupture, annulus rupture, or aortic dissection always required emergent surgery.

In general, bailout surgery after TAVR in the acute setting carries significant morbidity and mortality, and from our series, it is clear that not all complications have the same risk profile. Annular rupture carried a 0% survival rate in our small sample size, and even in centers with higher volumes of balloon-expandable devices, it is the most feared adverse event in the operating room. Moreover, due to their time-sensitive nature, acute aortic dissection and ventricular rupture can have potentially catastrophic consequences if not treated promptly. The mortality rate from our series, especially in the setting of acute complications, is in line with the numbers from other high-volume or multicenter registries [13,14]. One of the most recent multicenter analyses described an intraprocedural death rate of 12.5% and an in-hospital mortality rate of 49.3%. In regard to patients surviving index hospitalization, the one-year mortality rate was 15.3% [11]. Pineda et al. [13] demonstrated that the in-hospital mortality was in line with other registries and significantly higher in patients who required surgical bailout (49.64% vs. 3.52%; *p* < 0.0001) compared with patients who developed minor complications. It should not be understated that all patients undergoing TAVR at our institution have gone through a Heart Team discussion, and the decision for the transcatheter route is the only option when weighing the risk–benefit ratio for each patient. Moreover, a lot of TAVR candidates are deemed prohibitive surgical candidates, and this makes the potential bailout even more challenging, facing an already uphill battle in high-pressure and time-sensitive situations.

For both interventional cardiologists and CT surgeons, building extensive experience on the potential complications of TAVR and the relative bailout strategies is of paramount importance for minimizing morbidity and mortality. This allows, with time and growing expertise, the possibility to approach more complex patients that would otherwise be denied the optimal treatment strategy. This process of personalized treatment can only proceed safely in a setting where CT surgery is involved throughout the whole process of transcatheter valve procedures, from a Heart Team discussion to preoperative imaging and critical issues assessment prior to TAVR. Instituting heart valve centers with multidisciplinary teams composed of all the professionals involved in the management of patients with valve disease should be the goal in high-volume hospitals.

Because of the vastly increased risk associated with emergent or urgent surgery after TAVR, there needs to be an independent risk and prognostic model for this novel category of patients. This is particularly significant in a setting where the concept of life-long management of aortic valve disease in younger patients is becoming more and more relevant. Moreover, with individualized risk scores and preoperative assessment, there needs to be a process of consensus for the standardization of surgical approaches for patients who previously underwent transcatheter valve therapies. As TAVR becomes more and more widely adopted in a low-risk populations, we recommend a careful and open-minded Heart Team discussion in order to select the proper treatment for aortic valve disease and to anticipate the critical issues that may require a bailout strategy in order to avoid any futile decision when catastrophic scenarios occur.

The major limitations of our population and of the present study respectively are the small sample size, despite the long timeframe, and the paucity of adverse events—even with such numerous procedures performed. For this reason, the outcomes that have or have not reached statistical significance should be placed in the context of a single-center analysis with a very low rate of complications. The take-home message from this is that increasing surgical expertise and the volume of procedures minimizes complications and improves patient outcomes. Further multicenter studies and comprehensive registries are warranted to better clarify the topic and provide greater statistical power to our conclusions, with emphasis not only on complications and surgical bailout in the acute setting but also on the incidence and management of short-term complications of transcatheter valve treatment.

## 5. Conclusions

Bailout and post-TAVR surgery have become critical issues, especially with more and more devices being implanted in younger and lower-risk individuals, with overall acceptable yet significant mortality considering the very high risk of these procedures, thereby underlining the importance of having skilled CT surgery teams on site in all TAVR centers to promptly provide a resolution to scenarios such as repair of a ventricular perforation and the need for emergency aortic valve/root replacement. It is well established that self-expanding and balloon-expandable devices carry different risks and potential complications. Moreover, from our series it appears that catastrophic complications (such as annular rupture with hemodynamic collapse) may be associated with higher mortality for balloon-expandable valves; nevertheless, larger study series and multicenter registries are needed to provide validation.

## Figures and Tables

**Table 1:** Patient characteristics and results.

Demographics	Overall Population *n* = 21	Chronic Complications *n* = 10	Acute Complications *n* = 11	*p*-Value	Self-Expanding TAVR *n* = 16	Balloon-Expandable TAVR *n* = 5	*p*-Value
Median age [y] (IQR)	78 (11)	75 (11)	80 (7)	0.33	79 (12)	75 (9)	0.67
Female (%)	12 (57)	6 (60)	6 (54)	0.57	10 (63)	2 (40)	0.62
Diagnosis (%)							
Aortic stenosis	14 (67)	6 (60)	8 (73)	0.66	10 (63)	4 (80)	0.62
Aortic insufficiency	3 (14)	1 (10)	2 (18)	1	2 (12)	1 (20)	1
Valve-in-valve	4 (19)	3 (30)	1 (9)	0.31	4 (25)	0 (0)	0.54
Preoperative characteristics							
Mean BSA [m^2^] (SD)	1.8 (0.27)	1.82 (0.4)	1.78 (0.3)	0.79	1.81 (0.5)	1.76 (0.4)	0.84
Prior cardiac surgery (%)	5 (24)	2 (20)	3 (27)	0.67	4 (20)	1 (20)	1
Diabetes (%)	4 (19)	2 (20)	2 (18)	1	3 (18)	1 (20)	1
Hypertension (%)	14 (67)	6 (60)	8 (72)	0.45	10 (63)	4 (80)	0.62
Hyperlipidemia (%)	7 (33)	3 (30)	4 (36)	0.74	5 (31)	2 (40)	1
CHF (%)	4 (19)	2 (20)	2 (18)	1	2 (13)	2 (40)	0.23
Severe COPD (%)	2 (9.5)	0 (0)	2 (18)	0.22	2 (13)	0 (0)	1
Median creatinine [mg/dL] (IQR)	0.9 (0.7)	0.8 (0.4)	1.2 (0.5)	0.75	0.9 (0.6)	1 (0.6)	0.75
Mean LV ejection fraction [%] (SD)	58 (7.8)	59 (6.7)	56 (9.3)	0.68	57 (8.8)	58 (6.2)	0.82
Mean gradient [mmHg] (SD)	40.6 (19.1)	40 (17)	43 (22)	0.82	41.1 (19)	38.6 (25)	0.81
Median STS score (IQR)	2.4 (2.9)	3.2 (2.9)	2.3 (3.1)	0.67	2.4 (2.8)	2.3 (4.5)	0.95

List of abbreviations: IQR = interquartile range, CHF = chronic heart failure, STS = Society of Thoracic Surgeons, TAVR = transcatheter aortic valve replacement, and LV = left ventricle.

**Table 2:** Perioperative results.

	Overall Population *n* = 21	Chronic Complications *n* = 10	Acute Complications *n* = 11	*p*-Value	Self-Expanding TAVR *n* = 16	Balloon-Expandable TAVR *n* = 5	*p*-Value
Indication for TAVR (%)							
Age	14 (67)	5 (50)	9 (82)	0.18	10 (63)	4 (80)	0.62
Frailty/Risk	7 (33)	5 (50)	2 (18)	0.18	6 (37)	1 (20)	0.62
TAVR type (%)							
Self-expanding	16 (76)	7 (70)	9 (82)	0.64	-	-	
Balloon-expandable	5 (24)	3 (30)	2 (18)	0.64	-	-	
Complication timing (%)							
Acute	11 (52)	-	-		9 (56)	2 (40)	0.64
Chronic	10 (48)	-	-		7 (44)	3 (60)	0.64
Complication type (%)							
Displacement	1 (5)	0	1 (9)	-	1 (6)	0 (0)	1
Aortic dissection	4 (19)	0	4 (36)	-	4 (25)	0 (0)	0.53
Annulus rupture	2 (10)	0	2 (18)	-	0 (0)	2 (40)	0.05
LV perforation	3 (14)	0	3 (27)	-	3 (19)	0 (0)	0.55
Valve dysfunction	3 (19)	3 (30)	1 (9)	0.31	2 (12)	2 (40)	0.23
Endocarditis	5 (24)	5 (50)	0	-	4 (25)	1 (20)	1
Subclinical valve thrombosis/Significant PVL	2 (10)	2 (20)	0	-	2 (12)	0 (0)	1
Bailout strategy (%)							
Repair	4 (19)	0	4 (36)	0.09	4 (25)	0 (0)	0.53
Aortic valve replacement	10 (48)	7 (70)	3 (27)	0.09	6 (37)	4 (80)	0.15
Aortic valve + ascending aorta replacement	3 (14)	1 (10)	2 (18)	1	3 (19)	0 (0)	0.55
Aortic root replacement	4 (19)	2 (20)	2 (18)	1	3 (19)	1 (20)	1
In-hospital mortality (%)	6 (29)	2 (20)	4 (36)	0.64	3 (19)	3 (60)	0.11

List of abbreviations: TAVR = transcatheter aortic valve replacement, LV = left ventricle, and PVL = perivalvular leak.

## Data Availability

The data presented in this study are available on request to the corresponding author due to privacy issues.
